# Landmark Evolutions in Time and Indication for Cardiac Resynchronization Therapy: Results from a Multicenter Retrospective Registry

**DOI:** 10.3390/jcm13071903

**Published:** 2024-03-25

**Authors:** Jeroen Bijnens, Sander Trenson, Gabor Voros, Pieter Martens, Sebastian Ingelaere, Pascal Betschart, Jens-Uwe Voigt, Matthias Dupont, Alexander Breitenstein, Jan Steffel, Rik Willems, Frank Ruschitzka, Wilfried Mullens, Stephan Winnik, Bert Vandenberk

**Affiliations:** 1 Department of Cardiology, University Hospitals Leuven, 3000 Leuven, Belgiumgabor.voros@uzleuven.be (G.V.);; 2 Department of Cardiology, Sint-Jan Hospital Bruges, 8000 Bruges, Belgium; 3 Department of Cardiology, University Hospital Zurich, 8091 Zurich, Switzerland; 4 Department of Cardiovascular Sciences, KU Leuven, 3000 Leuven, Belgium; 5 Department of Cardiology, Ziekenhuis Oost-Limburg, 3600 Genk, Belgiummatthiasdupont@hotmail.com (M.D.);; 6 Hirslanden Heart Clinic, 8008 Zurich, Switzerland; 7 Department of Life Sciences, Hasselt University, 3500 Hasselt, Belgium; 8 Zurich Regional Health Center Wetzikon, 8620 Zurich, Switzerland

**Keywords:** cardiac resynchronization therapy, electrophysiology, heart failure, implantable, pacemaker

## Abstract

**Background:** Cardiac resynchronization therapy (CRT) has evolved into an established therapy for patients with chronic heart failure and a wide QRS complex. Data on long-term outcomes over time are scarce and the criteria for implantation remain a subject of investigation. **Methods:** An international, multicenter, retrospective registry includes 2275 patients who received CRT between 30 November 2000 and 31 December 2019, with a mean follow-up of 3.6 ± 2.7 years. Four time periods were defined, based on landmark trials and guidelines. The combined endpoint was a composite of all-cause mortality, heart transplantation, or left ventricular assist device implantation. **Results:** The composite endpoint occurred in 656 patients (29.2%). The mean annual implantation rate tripled from 31.5 ± 17.4/year in the first period to 107.4 ± 62.4/year in the last period. In the adjusted Cox regression analysis, the hazard ratio for the composite endpoint was not statistically different between time periods. When compared to sinus rhythm with left bundle branch block (LBBB), a non-LBBB conduction pattern (sinus rhythm: HR 1.51, 95% CI 1.12–2.03; atrial fibrillation: HR 2.08, 95% CI 1.30–3.33) and a QRS duration below 130 ms (HR 1.64, 95% CI 1.29–2.09) were associated with a higher hazard ratio. **Conclusions:** Despite innovations, an adjusted regression analysis revealed stable overall survival over time, which can at least partially be explained by a shift in patient characteristics.

## 1. Introduction

Over the past twenty years, cardiac resynchronization therapy (CRT) has evolved as one of the standards of care for patients with heart failure (HF), with a reduced ejection fraction (HFrEF) and a wide QRS complex [1,2]. In the early 2000s, several landmark trials demonstrated the clinical benefit of CRT in patients with HFrEF [3]. COMPANION [4], CARE-HF [5], and RAFT [6] proved the efficacy of CRT in patients with NYHA class III and IV. In 2008 and 2009, REVERSE [7] and MADIT-CRT [8] also revealed that patients with NYHA class I and II were likely to benefit from CRT. Since then, the integration of CRT into numerous clinical practice guidelines, both for pacing and for heart failure, has further underscored its significance.

In parallel, substantial progress has been made both in regard to technical aspects of CRT and pharmacological treatments for heart failure. Likewise, patient selection and implantation indications have evolved over time as new studies have been conducted. Nevertheless, data on how this historical evolution has affected patient selection, CRT implantation rates, and long-term outcomes are scarce.

Therefore, we aim to describe the evolution of patient characteristics, indications, and long-term outcomes in CRT patients over two decades of change and development, using a large, multicenter European CRT registry [9].

## 2. Materials and Methods

### 2.1. Study Population

All patients aged ≥18 years who had a CRT implantation in one of 3 participating tertiary care centers, University Hospital Zurich (Zurich, Switzerland), Ziekenhuis Oost-Limburg (Genk, Belgium), and University Hospitals Leuven (Leuven, Belgium), were included in a retrospective registry. The registry has been described previously [9]. In brief, indications for CRT implantation followed the latest literature or ESC guidelines available at the time [1,10]. Accordingly, ischemic, as well as non-ischemic, cardiomyopathies were included and the devices implanted had a cardioverter–defibrillation function, if indicated. Likewise, devices were implanted de novo, or were upgraded from a pacemaker or implantable cardioverter defibrillator (ICD). Optimization of the guideline-directed medical therapy and CRT programming were left to the discretion of the treating physicians. In general, each center had a routine follow-up routine, which included in-person visits and remote monitoring. Ethical approval was granted by the ethical committee at each individual institution. Given the retrospective nature of the study, the requirement for written informed consent was exempted.

### 2.2. Retrospective Registries

Every patient implanted with a CRT device between 30 November 2000 and 31 December 2019 was included in the registry. Inclusion dates and follow-up times differed between the three hospitals. Demographic data, clinical characteristics, and the baseline pharmaceutical regimen, alongside the biochemical, electrocardiographic, and echocardiographic information prior to CRT implantation were extracted from the electronic medical records. The left ventricular ejection fraction (LVEF) before CRT implantation was acquired from cardiac magnetic resonance imaging or echocardiography. For the latter, measurements were obtained using the modified Simpson’s biplane method or by visual assessment. The consolidation of registries was executed under the oversight of two investigators (B.V. and S.T.). In this study, exclusively shared variables were selected for subsequent analysis.

### 2.3. Arrangement by Time Periods and Indications

Four distinct time periods were identified, and each patient was allocated to one period according to their implantation date. The demarcation of these periods was chosen according to publication of landmark trials and guidelines. The first period (P1) ranged from the start of the registry (30 November 2000) to the publication of the MADIT-CRT findings on 1 October 2009. The subsequent period (P2) extended from the release of MADIT-CRT until the 2013 ESC pacing and CRT guidelines (25 June 2013). The third period (P3) covered the period from the publication of the 2013 ESC guidelines to the publication of the 2016 ESC guidelines on heart failure on 20 May 2016. The fourth, and last period, (P4) encompassed the time span from the 2016 ESC guidelines to the last patient included on 31 December 2019.

Additionally, analysis was performed according to the rhythm and QRS morphology in patients with a LVEF ≤ 35%. For this analysis, baseline data was arranged according to the presence of sinus rhythm or atrial fibrillation, related to distinctive conduction patterns, specifically left bundle branch block (LBBB) and non-left bundle branch block, and QRS duration > 150 milliseconds (ms), 150 ms to 130 ms and ≤130 ms.

### 2.4. Endpoints

The study endpoint was a composite of left ventricular assist device implantation, heart transplantation, or all-cause mortality. Specific endpoint occurrences were documented alongside the corresponding dates. Patients who did not experience the composite endpoint were included in the analysis from the date of implantation until the most recent available follow-up, defined as the last clinical contact. If the patient experienced the composite endpoint, the date of decease, heart transplantation, or left ventricular assist device implantation, was considered the last follow-up date. Patients from Ziekenhuis Oost-Limburg who required transplantation or ventricular assist device implantation were referred to University Hospitals Leuven.

### 2.5. Statistical Analysis

Categorical variables were presented as numbers and percentages. The normal distribution of continuous variables was verified using the Kolmogorov–Smirnov test. Since all continuous variables showed non-normal distribution, these were presented as median and interquartile ranges. A comparison of the parameters between groups was performed using the Kruskal–Wallis test, the Mann–Whitney U test, and the chi-square test. Given the differences in inclusion between the centers, implant rates were adjusted according to the available time in each period and expressed as the mean annual implant rate. Implant rates were calculated separately for each center and analyzed using the chi-square test for any trends. Kaplan–Meier analysis was performed to calculate incidence rates for the composite endpoint, including the log-rank test for comparison by group. Univariable and multivariable Cox proportional hazards regression modelling was performed for the combined endpoint. A multivariable model for the combined endpoint was constructed by including all variables with a *p*-value < 0.100 in the univariable Cox regression, in a stepwise multivariable model with forward parameter selection (entry *p* < 0.050). The proportional hazard assumptions were assessed using the Schoenfeld residuals test and proportional hazard plots, while multicollinearity was assessed using covariance matrices. In case of violation of the proportional hazard assumptions, stratification was applied. Next, for the adjusted analysis according to the implant period and rhythm and QRS morphology, these variables were added to the previously developed multivariable Cox regression model. For the analysis according to the rhythm and QRS morphology, variables which included information about the QRS duration and QRS morphology were removed from the previously developed model. For each model, the *p*-value corresponding to the global Schoenfeld residuals test and Harrell’s C-index are reported either in the manuscript or in the Supplementary Materials. An overview of the data availability is presented in Supplementary Table S1. Missing values were handled by listwise deletion. The statistical analyses were performed using Stata version 17.0 (StataCorp LLC, College Station, TX, USA) and GraphPad Prism version 9.00 (GraphPad Software, La Jolla, CA, USA).

## 3. Results

### 3.1. Demographics

A total of 2275 patients were enrolled in the registry and the data availability was excellent, with a maximum of 2.3% missing data for all variables (Supplementary Table S1). The baseline demographic characteristics and a comparison between the four time periods are shown in Table 1. In general, the median age at implantation was 70.3 years and 26.4% were female. In 63.9% of patients, an implantable cardioverter defibrillator was implanted and, in 42.8%, the underlying cause of heart failure was an ischemic cardiomyopathy (ICMP). Overall, two-thirds (66.8%) of cases showed left bundle branch block (LBBB) at the time of device implantation, while the remaining portion exhibited non-LBBB conduction patterns. Notably, in 18.9% of implantations, the QRS complex was below 130 ms.

### 3.2. Endpoint Analysis

**Figure 1:**
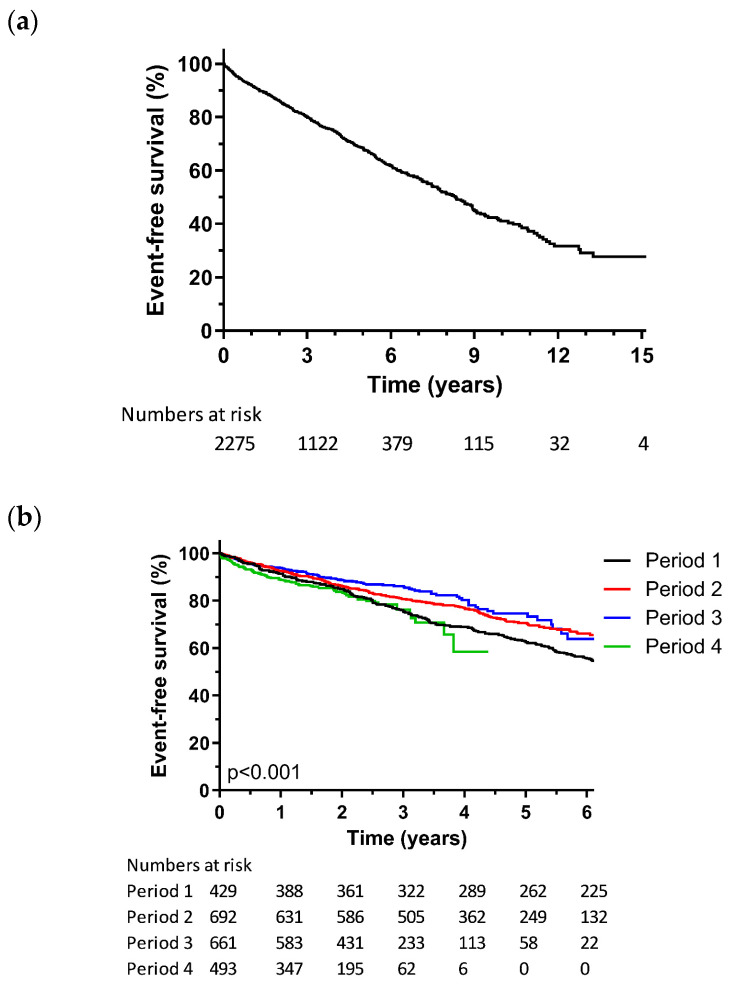

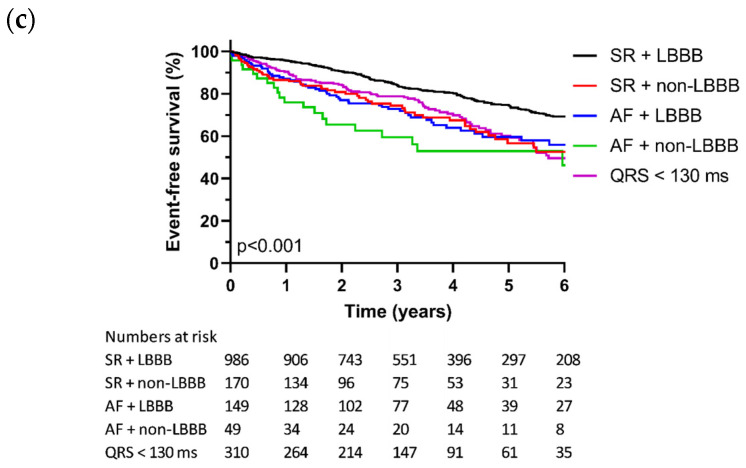
Kaplan–Meier analysis for all-cause mortality, heart transplantation, or ventricular assist device; (**a**) Kaplan–Meier analysis for the overall population; (**b**) Kaplan–Meier analysis according to implant period; (**c**) Kaplan–Meier analysis according to rhythm and QRS morphology.

The primary composite endpoint occurred in 656 patients (29.2%). Of these, 34 underwent ventricular assist device implantation, 37 underwent heart transplantation, and 585 patients died. The mean follow-up was 3.6 ± 2.7 years. The Kaplan–Meier analysis conducted for the composite endpoint in the overall population is shown in Figure 1A.

Table 2 shows the multivariable Cox proportional hazards regression model for endpoint occurrences in the overall registry. Independent predictors for the composite endpoint were male sex, no ICD, a QRS duration below 130 ms, a non-LBBB conduction pattern, and the absence of pharmacological RAAS inhibition. On the other hand, a lower NYHA functional class, better renal function, the absence of diabetes mellitus, and no history of stroke, or transient ischemic attack, were independently associated with a protective effect with regard to the composite endpoint.

### 3.3. Evolution in Time

**Figure 2:**
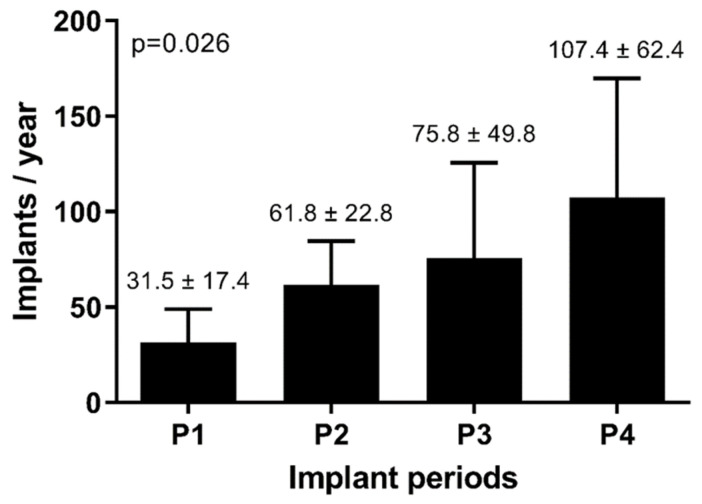
Evolution of mean implant rate.

Progression of the mean annual implant rate across the four distinct periods is demonstrated in Figure 2. Notably, there was a significant and consistent upward trajectory in the number of annual CRT implantations (*p* = 0.026). Table 1 provides an overview of the evolution of the patient characteristics throughout the four periods, with group-to-group comparisons available in Supplementary Table S2. Over the course of the last two decades, patients who underwent CRT implantation have shown a trend toward older ages. The implantation of combined CRT–ICD devices (CRT-D) was higher in the first period compared to subsequent periods. Likewise, the implantation rates have shifted to lower NYHA functional classes in the latter three periods compared to the initial period. In addition, there has been a trend toward decreased implantation of epicardial leads over time.

In our registry, conduction patterns varied as the indication was not confined solely to left bundle branch block patterns. Right bundle branch block patterns, non-specific patterns, and paced QRS patterns were observed with varying frequency over the distinct periods. In the final period, a lower proportion of patients were on renin–angiotensin–aldosterone system (RAAS) inhibitors before implantation than in the three first periods. The rate of patients taking loop diuretics displayed an initial downward trend, but increased significantly in the last period.

### 3.4. Endpoint Prediction over Time

The Kaplan–Meier survival curves for the composite endpoint across consecutive time periods are demonstrated in Figure 1B. As shown in Table 3, the cumulative event rates at the 1-year follow-up for P1, P2, P3, and P4 were 8.5%, 7.5%, 6.1%, and 11%, respectively. Over a 3-year span, the cumulative event rates of 24.5%, 19.4%, 13.8%, and 23.8% were observed, and 5-year cumulative event rates of 37.6%, 29.3%, and 25.4% were observed, with 5-year follow-up data not available for P4. In an adjusted Cox proportional hazards regression analysis, the hazard ratio for the composite endpoint was not statistically different between the four periods (Table 3). The final Cox proportional hazards regression model, including analysis by implant period, is presented in Supplementary Table S3.

### 3.5. Endpoint Prediction According to Rhythm and QRS Morphology

The baseline demographics and clinical characteristics arranged according to the rhythm and QRS morphology are shown in Table 4. Sinus rhythm in combination with an LBBB conduction pattern was significantly more prevalent in women compared to men, whereas the prevalence of sinus rhythm with different conduction patterns or atrial fibrillation with LBBB, non-LBBB, or a QRS below 130 ms, displayed a more balanced distribution between the sexes. Other differences are described in Supplementary Table S4, but they lack meaningful correlations.

The Kaplan–Meier analysis for the composite endpoint, broken down by rhythm and QRS morphology, is displayed in Figure 1C. The cumulative event rate after 5 years was 25.6% for sinus rhythm with an LBBB conduction pattern, while it ranged from 39.8% to 47.1% for patients who had atrial fibrillation and/or non-LBBB conduction patterns (Table 5, Supplementary Table S5). In an adjusted Cox proportional hazards regression model (Table 5), the presence of a non-LBBB conduction pattern was associated with a higher hazard ratio in regard to both sinus rhythm (HR 1.51, 95% CI 1.12–2.03) and atrial fibrillation (HR 2.08, 95% CI 1.30–3.33) for the composite endpoint, compared to an LBBB conduction pattern and sinus rhythm (reference group). Likewise, a QRS duration below 130 ms exhibited a higher hazard ratio (HR 1.64, 95% CI 1.29–2.09) compared to an LBBB conduction pattern and sinus rhythm. There was no significant difference between the patients with LBBB in regard to sinus rhythm and patients with LBBB in regard to AF (HR 1.33, 95% CI 0.97–1.80). The final Cox regression model is presented in Supplementary Table S6.

## 4. Discussion

In this large, multicenter, retrospective CRT registry, representing the real-world experience of three tertiary care centers, we examined the evolution of CRT implantations over time and explored the different implant indications, patient populations, and outcomes.

### 4.1. Evolution over Time

Over the last two decades, CRT has become one of the cornerstones of treatment in HFrEF, specifically in patients with conduction delays. This is illustrated by the increment in implantations over the years, as the total number of annual CRT implantations has more than tripled from the first period to the fourth period. This increase reflects a combination of the increasing prevalence of heart failure and the evolving indications for CRT. The temporal shift in patient selection criteria is reflected in the baseline clinical characteristics. For example, the observed shift in annual implant rates and NYHA class, with a higher prevalence of NYHA class III in P1, reflects the findings in the MADIT-CRT study, which primarily focused on patients with moderate-to-severe heart failure [8]. This expanded the potential pool of CRT candidates to a different subset of the patient population, with potentially different disease characteristics. Further, CRT devices have been increasingly implanted in older patients, leading to a higher burden from comorbidities and, consequently, an elevated likelihood of reaching the composite endpoint, irrespective of their comparable improvement in left ventricular function, as a recent retrospective analysis in 2656 geriatric patients showed [11]. Also, the rate of implanted epicardial leads declined over time. This decline may be attributed to technological evolutions, such as quadripolar leads [12,13] and active fixation techniques [14], which have resulted in more stable transvenous pacing. Additionally, the decline in the implantation of a device with a defibrillator function over time, might be attributed to the more stringent criteria for ICD implantation in increasing age, as in our registry almost 25% of patients were 77 years or older, as well as the lack of randomized data indicating a clear benefit of CRT-D over CRT-P [15].

While unadjusted cumulative event rates in our registry declined over time, apart from P4 which extends over the COVID-19 era, these findings did not persist in the adjusted regression analysis. The overall event-free rate aligns with the recent long-term follow-up of the RAFT study, which included patients in NYHA class II and III, who also constitute the predominant portion of our registry [16]. The 5-year cumulative event-rate for the similar composite endpoint in RAFT, based on a visual assessment of the Kaplan–Meier graphs, was approximately 30% and is, therefore, comparable with our multicenter real-world experience of 31.8% [16]. Real-world data on the evolution of mortality and the outcome of CRT are scarce so far, with only two recent analyses of retrospective registries. Darden et al. described a United States registry, from 2011 to 2015, of patients aged ≥65 years implanted with a CRT-D, which disclosed a lower mortality at the 2-year follow-up [17]. Similarly, another retrospective registry, by Leyva et al., encompassing data from 2010 to 2019 from the United Kingdom, also showed decreased mortality after the 2-year follow-up period, with a hazard ratio of 0.72 (95% CI 0.69–0.76) when comparing 2010–2011 to 2018–2019 [18], despite demonstrating increasing comorbidities. While our analysis may be prone to the effect of unknown or unavailable confounders, as are all retrospective studies, there are relevant differences between our registry and previous registries, which may in part be responsible for the difference in outcome. Besides the age cut-off, the study by Darden et al. only included patients who underwent de novo CRT-D implantations and the use of ACE inhibitors and angiotensin-receptor blockers was approximately 76%. Also, the study by Leyva et al. did not include patients who underwent a CRT upgrade [9] and the data availability was at a different level of granularity (e.g., chronic kidney disease as a binary variable versus different chronic kidney disease stages in our study). On the other hand, we did not have detailed information available on the peri- or postprocedural complications, which may have had an influence given the increasing frailty of the patients. Of note, data from our registry predates the widespread use of sodium–glucose cotransporter 2 inhibitors for heart failure, while angiotensin receptor neprilysin inhibitors [19] were only introduced briefly before the inclusion of the last patient.

### 4.2. Implant Indication

The current cardiac pacing and CRT guidelines [1] suggest the strongest response as CRT for patients with a QRS duration exceeding 150ms and an LBBB conduction pattern. However, the guidelines also advocate, albeit with a less robust recommendation, for the implantation of CRT devices in patients with a QRS duration ≥ 150 ms and a non-LBBB conduction pattern, and for a QRS between 130–150 ms and an LBBB conduction pattern. An even weaker recommendation for CRT is formulated for cases with a QRS duration between 130–150 ms and a non-LBBB conduction pattern.

Electrophysiological properties that predict a favorable response to CRT remain a topic of ongoing debate. No randomized controlled trials (RCTs) have been performed specifically to assess disease modification by CRT in different QRS morphologies. Yet a substudy of RAFT [20], showed a benefit from CRT in patients with an LBBB conduction pattern and a QRS ≥ 160 ms, which appeared to be absent in patients with a non-LBBB conduction pattern, especially if the QRS was below 160 ms. A similar conclusion was drawn from a post hoc analysis of MADIT-CRT [21]. A post hoc meta-analysis of five RCTs confirmed this correlation between an LBBB conduction pattern and a more beneficial response to CRT [22], while there remains more ambiguity regarding patients with non-LBBB conduction patterns [23]. Other meta-analyses place greater emphasis on the QRS duration, suggesting patients with a QRS duration below 150 ms appear to derive less benefits from CRT implantation, irrespective of the conduction pattern [24,25].

Within our multicenter real-world registry, although the non-adjusted incidence rates for the composite endpoint were comparable between patients with a QRS duration of ≥150 ms and patients with a QRS duration between 130 and 150 ms (Supplementary Table S5), the final adjusted model revealed a significant difference only in regard to the conduction pattern. Specifically, patients with an LBBB conduction pattern had a lower chance of reaching the composite endpoint when compared to patients with a non-LBBB conduction pattern, irrespective of the QRS duration at the time of implantation (Supplementary Table S5). Considering the absence of true control groups without CRT, we are restricted to reporting this observation only. Early retrospective studies have also indicated increased event rates [26,27] or less echocardiographic and symptomatic benefit [28] after a 3-year follow-up period among patients with a non-LBBB conduction pattern. Importantly, our study does not indicate a non-response in regard to CRT among patients with a non-LBBB conduction pattern.

The inquiry into which patients, specifically which QRS duration and conduction pattern, benefit the most from CRT implantation remains a subject of research, partly because in most meta-analyses non-LBBB conduction patterns are mostly aggregated into one group. A recent meta-analysis advocates subdivision within the non-LBBB category into more specific groups, such as intraventricular conduction delay (IVCD) and right bundle branch block (RBBB) [29]. It suggests that patients with a QRS duration of longer than 150 ms and IVCD benefit more from CRT than patients with a QRS ≥ 150 ms and RBBB. Our registry recorded 18.9% of implantations in patients with a QRS duration below 130 ms, despite findings from EchoCRT [30] indicating no benefit, and possibly even harm, from CRT in this population. This number is comparable to the number of guideline-discordant implantations found in the registry by Darden et al., but may reflect true BLOCK-HF patients [17,31].

Additionally, it is important to acknowledge that our registry is influenced by temporal changes in the guidelines. This, in particular, has an effect on the interpretation of implant indication. For instance, the definition of left bundle branch block, according to the ESC guidelines, was subject to change over the years, leading to different patient selection and potentially effecting the interpretation of clinical endpoints in disease modification by CRT [32].

### 4.3. Limitations

The retrospective study design is associated with the inherent limitations of retrospective research, including the potential impact of missing data. Given the absence of a true control population, we are unable to define absolute clinical benefits and we are limited to indirect observations only. As such, our study only reports associations without implying causality for the observed associations. The analysis was constrained by the restricted set of biochemical and echocardiographic variables, encompassing only those available for all patients among the participating centers. Therefore, several variables of interest were not available for analysis, including QRS duration after CRT implantation, atrioventricular conduction delays, and left ventricular remodeling variables, such as the left ventricular end-diastolic diameter. Lastly, the primary endpoint was a composite of endpoints encompassing all-cause mortality, heart transplantation, and implantation of ventricular assist device. As such, it does not include data about changes in quality of life, heart failure admission rates, or functional improvement. Retrospective ascertainment of these potential endpoints imposes a risk of bias to the analysis.

## 5. Conclusions

This real-world registry of 2275 patients presents the evolution of CRT implantations over almost two decades, showing a notable increase in procedures. Despite pharmaceutical and technological innovations, an adjusted regression analysis revealed stable overall survival over time, at least partially explained by the shift in patient characteristics. These findings highlight the challenges to the ongoing quest to improve clinical outcomes.

## Figures and Tables

**Table 1:** Baseline demographics and clinical characteristics by implant period.

Variable	All Patients	Period 1	Period 2	Period 3	Period 4	*p*-Value
N	2275 (100%)	429 (18.9%)	692 (30.4%)	661 (29.1%)	493 (21.7%)	
Age implant (y)	70.3 (61.8–76.8)	67.1 (58.8–73.4)	69.5 (62.3–76.4)	72.7 (64.7–78.6)	71.1 (62.8–78.1)	<0.001
Female	596 (26.4%)	97 (22.6%)	194 (28.1%)	192 (29.2%)	113 (23.3%)	0.026
ICD	1452 (63.9%)	312 (72.9%)	437 (63.2%)	387 (58.6%)	316 (64.4%)	<0.001
Upgrade	605 (26.6%)	114 (26.6%)	174 (25.1%)	160 (24.2%)	157 (32.0%)	0.019
Epicardial	136 (6.0%)	37 (8.6%)	45 (6.5%)	32 (4.9%)	22 (4.5%)	0.028
ICMP	962 (42.8%)	211 (49.4%)	303 (43.9%)	255 (38.9%)	193 (40.6%)	0.005
LVEF (%)	27.0 (21.0–34.0)	25.0 (20.0–30.0)	28.0 (22.9–35.0)	28.0 (22.0–35.0)	29.0 (22.0–34.3)	<0.001
LVEF ≤ 35%	1890 (84.9%)	386 (90.8%)	578 (84.3%)	536 (83.0%)	390 (83.0%)	0.002
NYHA						
I	86 (3.9%)	11 (2.6%)	28 (4.1%)	22 (3.4%)	25 (5.4%)	<0.001
II	695 (31.3%)	76 (17.8%)	231 (33.5%)	216 (33.5%)	172 (37.1%)	
III	1350 (60.7%)	308 (72.3%)	402 (58.4%)	392 (60.9%)	248 (53.5%)	
IV	92 (4.1%)	31 (7.3%)	28 (4.1%)	14 (2.2%)	19 (4.1%)	
eGFR (mL/min)	57.8 (41.2–74.7)	56.0 (40.9–71.8)	59.8 (41.3–76.2)	59.9 (41.4–75.5)	54.3 (40.2–72.5)	0.065
CKD 1-2	1043 (47.1%)	186 (43.6%)	334 (49.6%)	317 (49.8%)	206 (42.9%)	0.178
CKD 3a	489 (22.1%)	104 (24.4%)	141 (21.0%)	128 (20.1%)	116 (24.2%)	
CKD 3b	401 (18.1%)	86 (20.1%)	121 (18.0%)	106 (16.6%)	88 (18.3%)	
CKD 4-5	284 (12.8%)	51 (11.9%)	77 (11.4%)	86 (13.5%)	70 (14.6%)	
QRS duration (ms)	158 (138–176)	162 (138–182)	160 (140–178)	156 (136–172)	158 (140–174)	0.003
≤130 ms	420 (18.9%)	76 (18.1%)	130 (18.9%)	131 (20.3%)	83 (17.5%)	0.204
130–150 ms	487 (21.9%)	79 (18.9%)	140 (20.4%)	154 (23.9%)	114 (24.0%)	
>150 ms	1319 (59.3%)	264 (63.0%)	417 (60.7%)	360 (55.8%)	278 (58.5%)	
Ventricular conduction						
Normal	175 (7.8%)	28 (6.7%)	55 (8.0%)	53 (8.2%)	39 (8.2%)	<0.001
RBBB	198 (8.9%)	27 (6.4%)	63 (9.2%)	71 (10.9%)	37 (7.8%)	
LBBB	1493 (66.8%)	285 (67.7%)	472 (68.6%)	459 (70.7%)	277 (58.2%)	
Unspecific	193 (8.6%)	33 (7.8%)	47 (6.8%)	35 (5.4%)	78 (16.4%)	
Paced	175 (7.8%)	48 (11.4%)	51 (7.4%)	31 (4.8%)	45 (9.5%)	
Rhythm						
Sinus	1680 (75.3%)	319 (75.4%)	514 (74.7%)	514 (79.7%)	333 (70.0%)	<0.001
AF	391 (17.5%)	62 (14.7%)	127 (18.5%)	108 (16.7%)	94 (19.8%)	
Atrial pacing	161 (7.2%)	42 (9.9%)	47 (6.8%)	23 (3.6%)	49 (10.3%)	
ACE/ARB/ARNI	1945 (86.2%)	393 (91.6%)	606 (88.0%)	571 (87.0%)	375 (77.6%)	<0.001
BB	1919 (85.0%)	366 (85.3%)	596 (86.5%)	549 (83.7%)	408 (84.5%)	0.523
MRA	1368 (60.6%)	245 (57.1%)	434 (63.1%)	397 (60.5%)	292 (60.5%)	0.265
Loop diuretic	1419 (63.2%)	328 (78.1%)	441 (64.0%)	348 (53.3%)	302 (62.7%)	<0.001
Amiodarone	514 (22.8%)	120 (28.0%)	144 (21.0%)	142 (21.7%)	108 (22.4%)	0.041
Hypertension	1535 (68.0%)	239 (55.7%)	447 (64.7%)	522 (79.7%)	327 (67.7%)	<0.001
Dyslipidemia	1451 (64.5%)	266 (62.2%)	390 (56.7%)	467 (71.5%)	328 (68.1%)	<0.001
History of stroke	229 (10.2%)	49 (11.5%)	71 (10.3%)	55 (8.4%)	54 (11.4%)	0.287
Diabetes mellitus	602 (26.6%)	122 (26.1%)	174 (25.2%)	177 (27.0%)	139 (28.7%)	0.600

**Table 2:** Multivariable Cox proportional hazards regression model for all-cause mortality, heart transplantation, or ventricular assist device.

Variable	Hazard Ratio	95% CI	*p*-Value
Female	0.64	0.52–0.80	<0.001
ICD	0.66	0.55–0.79	<0.001
ICMP	1.29	1.09–1.54	0.003
LVEF (/%)	0.98	0.97–0.99	0.003
NYHA			
I	reference		
II	1.80	0.86–3.74	0.116
III or IV	2.09	1.02–4.30	0.045
Renal function			
CKD 1-2	reference		
CKD 3a	1.21	0.97–1.52	0.086
CKD 3b	1.66	1.33–2.07	<0.001
CKD 4-5	2.70	2.12–3.45	<0.001
QRS duration			
≤130 ms	reference		
130–150 ms	0.75	0.57–0.97	0.030
>150 ms	0.78	0.63–0.97	0.027
LBBB	0.73	0.62–0.87	0.001
ACE/ARB/ARNI	0.58	0.46–0.73	<0.001
Diabetes mellitus	1.21	1.02–1.44	0.029
Stroke/TIA	1.26	1.01–1.58	0.041

The model was stratified by implant center and use of loop diuretics due to violation of the Schoenfeld residuals. Global Schoenfeld residuals test of the final model: *p* = 0.227. Harrell’s C-index of the final model = 0.669.

**Table 3:** Adjusted Cox proportional hazards regression model for all-cause mortality, heart transplantation, or ventricular assist device, by implant period.

	Events (n,%)	Incidence Rate (%/y, 95% CI)	Cumulative Event Rate (%)			Cox Regression	
			1 y	3 y	5 y	Unadjusted	Adjusted
Mortality/HTX/VAD	656 (29.2%)	8.0% (7.4–8.6)	8.1%	20.0%	31.8%		
Period 1	260 (60.6%)	9.9% (8.7–11.1)	8.7%	24.5%	37.6%	reference	reference
Period 2	208 (30.4%)	7.0% (6.1–8.0)	7.5%	19.4%	29.3%	0.73 (0.60–0.88)	0.93 (0.75–1.14)
Period 3	105 (16.1%)	5.9% (4.9–7.2)	6.1%	13.8%	25.4%	0.64 (0.50–0.81)	0.87 (0.67–1.13)
Period 4	83 (17.2%)	9.9% (8.0–12.3)	11.0%	23.8%	NA	1.05 (0.80–1.38)	1.02 (0.76–1.37)

The final Cox regression model was adjusted for sex, ICD, etiology of cardiomyopathy, left ventricular ejection fraction, NYHA class, QRS duration, renal function, left bundle branch block, ACE-inhibitor/angiotensin-receptor blocker/angiotensin receptor neprilysin inhibitor, diabetes mellitus, and stroke. The analysis was stratified by implant center and use of loop diuretics. The final model is presented as Supplementary Table S3.

**Table 4:** Baseline demographics and clinical characteristics according to rhythm and QRS morphology in patients with LVEF ≤ 35%.

Variable	SR + LBBB	SR + Non-LBBB	AF + LBBB	AF + Non-LBBB	QRS < 130 ms	*p*-Value
N	986	170	149	49	310	
Age implant (y)	69.3 (60.9–75.8)	68.4 (59.7–75.3)	74.0 (67.4–80.2)	72.8 (66.5–80.0)	67.7 (59.6–75.0)	<0.001
Female	334 (33.7%)	24 (14.1%)	26 (17.3%)	5 (10.2%)	65 (20.8%)	<0.001
ICD	694 (70.1%)	132 (77.7%)	77 (51.3%)	32 (65.3%)	241 (77.2%)	<0.001
Upgrade	147 (14.9%)	58 (34.1%)	47 (31.3%)	15 (30.6%)	50 (16.0%)	<0001
Epicardial	47 (4.8%)	9 (5.3%)	10 (6.8%)	1 (2.0%)	12 (3.9%)	0.604
ICMP	364 (36.8%)	99 (58.6%)	73 (48.7%)	24 (49.0%)	157 (50.7%)	<0.001
LVEF (%)	25.0 (20.0–30.0)	25.0 (20.0–30.0	25.0 (20.0–30.0	29.0 (25.0–30.0)	25.0 (20.0–30.0	0.309
NYHA						
I	35 (3.6%)	9 (5.4%)	5 (3.4%)	0 (0.0%)	9 (2.9%)	0.214
II	293 (29.8%)	52 (51.1%)	32 (21.5%)	13 (27.7%)	91 (29.6%)	
III	621 (63.1%)	98 (58.7%)	100 (67.1%)	33 (77.2%)	191 (62.0%)	
IV	36 (3.7%)	8 (4.8%)	12 (8.1%)	1 (2.1%)	17 (5.5%)	
eGFR (mL/min)	60.6 (43.0–77.3)	55.0 (40.6–70.7)	48.7 (32.9–61.9)	47.2 (35.8–60.4)	59.6 (41.6–74.2)	<0.001
CKD 1-2	500 (51.3%)	72 (42.9%)	44 (29.5%)	13 (27.7%)	151 (49.4%)	<0.001
CKD 3a	201 (20.6%)	43 (25.6%)	40 (26.9%)	12 (25.5%)	68 (22.2%)	
CKD 3b	176 (18.1%)	31 (18.5%)	32 (21.5%)	16 (34.0%)	37 (12.1%)	
CKD 4-5	97 (10.0%)	22 (13.1%)	33 (22.2%)	6 (12.8%)	50 (16.3%)	
ACE/ARB/ARNI	886 (89.5%)	138 (81.2%)	122 (81.3%)	43 (87.8%)	277 (88.8%)	0.003
BB	865 (87.4%)	143 (84.1%)	130 (86.7%)	40 (81.6%)	276 (88.5%)	0.521
MRA	651 (65.8%)	105 (61.8%)	98 (65.3%)	23 (46.9%)	208 (66.7%)	0.075
Loop diuretics	594 (60.4%)	123 (72.4%)	113 (75.3%)	35 (71.4%)	217 (70.0%)	<0.001
Amiodarone	205 (20.7%)	55 (32.4%)	41 (27.3%)	11 (22.5%)	69 (22.2%)	0.011
Hypertension	635 (64.2%)	118 (69.4%)	120 (80.0%)	33 (67.4%)	211 (67.6%)	0.004
Dyslipidemia	613 (62.1%)	121 (71.2%)	105 (70.0%)	28 (57.1%)	198 (63.5%)	0.069
Stroke	102 (10.4%)	14 (8.2%)	23 (15.4%)	4 (8.2%)	19 (6.1%)	0.025
Diabetes mellitus	254 (25.7%)	57 (33.5%)	35 (23.3%)	15 (30.6%)	91 (29.2%)	0.155

**Table 5:** Adjusted Cox proportional hazards regression model for all-cause mortality, heart transplantation, or ventricular assist device, by indication.

Rhythm	Morphology	Events (n,%)		Incidence Rate (%/y, 95% CI)	Cumulative Event Rate (%)			Cox Regression	
					1 y	3 y	5 y	Unadjusted	Adjusted
SR	LBBB	986	246 (25.0%)	6.1% (5.3–6.9)	4.4%	16.0%	25.6%	reference	reference
SR	Non-LBBB	170	62 (36.5%)	11.8% (9.2–15.2)	13.5%	25.7%	43.3%	1.96 (1.49–2.60)	1.51 (1.12–2.03)
AF	LBBB	149	56 (37.6%)	10.7% (8.3–13.9)	13.0%	27.2%	40.3%	1.80 (1.34–2.41)	1.33 (0.97–1.80)
AF	Non-LBBB	49	23 (46.9%)	16.4% (10.8–25.0)	24.1%	40.6%	47.1%	2.76 (1.78–4.28)	2.08 (1.30–3.33)
QRS < 130 ms		310	100 (32.3%)	9.9% (8.1–12.1)	9.6%	21.2%	39.8%	1.68 (1.33–2.13)	1.64 (1.29–2.09)

The final Cox regression model was adjusted for sex, ICD, etiology of cardiomyopathy, left ventricular ejection fraction, NYHA class, renal function, ACE inhibitor/angiotensin-receptor blocker/angiotensin receptor neprilysin inhibitor, diabetes mellitus, and stroke. The analysis was stratified according to implant center and use of loop diuretics. The final model is presented as Supplementary Table S6.

## Data Availability

The data presented in this study are available on request from the corresponding author (accurately indicate status).

## Supplementary Materials

The following supporting information can be downloaded at: https://www.mdpi.com/article/10.3390/jcm13071903/s1, Table S1: Data availability; Table S2: Between group comparison of demographics and clinical characteristics for implant period; Table S3: Final Cox proportional hazard regression model for the combined endpoint by implant period; Table S4: Between group comparison of demographics and clinical characteristics by rhythm and QRS morphology; Table S5: Detailed incidence and cumulative event rates for the combined endpoint by rhythm and QRS morphology and duration in patients with LVEF ≤ 35%; Table S6: Final Cox proportional hazard regression model for the combined endpoint by rhythm and QRS morphology; Table S7: QRS duration categorized.
