# Supplementary material for: Landmark Evolutions in Time and Indication for Cardiac Resynchronization Therapy: Results from a Multicenter Retrospective Registry
Source: J Clin Med. 2024 Mar 25;13(7):1903. doi: 10.3390/jcm13071903 (PMC11012510; doi:10.3390/jcm13071903)
Supplement: Supplementary file 1 [file jcm-13-01903-s001.zip › jcm-2900696-supplementary.pdf]

Supplementary Table S1. Data availability.

Variable	Available data	
Total n	2275	
Age implant (y)	2274	99.96%
Sex	2262	99.42%
ICD	2271	99.82%
Upgrade	2275	100.00%
Epicardial	2254	99.08%
ICMP	2249	98.86%
LVEF (%)	2227	97.89%
NYHA class	2223	97.71%
eGFR (mL/min)	2217	97.45%
QRS duration (ms)	2226	97.85%
Ventricular conduction	2226	97.85%
Rhythm	2232	98.11%
ACE / ARB / ARNI	2257	99.21%
BB	2257	99.21%
MRA	2256	99.16%
Loop diuretic	2244	98.64%
Amiodarone	2252	98.99%
Hypertension	2258	99.25%
Dyslipidemia	2251	98.95%
History of stroke	2248	98.81%
Diabetes Mellitus	2261	99.38%
Composite endpoint	2250	98.90%

ICD–implantable cardioverter-defibrillator; ICMP–ischemic cardiomyopathy; LVEF–left ventricular ejection fraction

Supplementary Table S2. Between group comparison of demographics and clinical characteristics for implant period.

Variable	Period 1	Period 2	Period 3	Period 4	Overall p-value	Between group comparison*					
						P1 vs P2	P1 vs P3	P1 vs P4	P2 vs P3	P2 vs P4	P3 vs P4
N	429 (18.9%)	692 (30.4%)	661 (29.1%)	493 (21.7%)							
Age implant (y)	67.1 (58.8 – 73.4)	69.5 (62.3 – 76.4)	72.7 (64.7 – 78.6)	71.1 (62.8 – 78.1)	<0.001	***<0.001***	***<0.001***	***<0.001***	***<0.001***	0.064	0.051
Female	97 (22.6%)	194 (28.1%)	192 (29.2%)	113 (23.3%)	0.026	0.043	0.016	0.805	0.641	0.066	0.025
ICD	312 (72.9%)	437 (63.2%)	387 (58.6%)	316 (64.4%)	<0.001	***<0.001***	***<0.001***	***0.006***	0.089	0.670	0.049
Upgrade	114 (26.6%)	174 (25.1%)	160 (24.2%)	157 (32.0%)	0.019	0.595	0.379	0.080	0.689	0.011	***0.004***
Epicardial	37 (8.6%)	45 (6.5%)	32 (4.9%)	22 (4.5%)	0.028	0.107	0.010	0.019	0.279	0.337	0.977
ICMP	211 (49.4%)	303 (43.9%)	255 (38.9%)	193 (40.6%)	0.005	0.070	***<0.001***	***0.007***	0.067	0.262	0.584
LVEF (%)	25.0 (20.0 – 30.0)	28.0 (22.9 – 35.0)	28.0 (22.0 – 35.0)	29.0 (22.0 – 34.3)	<0.001	***<0.001***	***<0.001***	***<0.001***	0.570	0.604	0.888
LVEF ≤ 35%	386 (90.8%)	578 (84.3%)	536 (83.0%)	390 (83.0%)	0.002	***0.002***	***<0.001***	***<0.001***	0.527	0.583	0.998
NYHA											
I	11 (2.6%)	28 (4.1%)	22 (3.4%)	25 (5.4%)	<0.001	***<0.001***	***<0.001***	***<0.001***	0.212	0.357	0.025
II	76 (17.8%)	231 (33.5%)	216 (33.5%)	172 (37.1%)							
III	308 (72.3%)	402 (58.4%)	392 (60.9%)	248 (53.5%)							
IV	31 (7.3%)	28 (4.1%)	14 (2.2%)	19 (4.1%							
eGFR (mL/min)	56.0 (40.9 – 71.8)	59.8 (41.3 – 76.2)	59.9 (41.4 – 75.5)	54.3 (40.2 – 72.5)	0.065						
CKD 1-2	186 (43.6%)	334 (49.6%)	317 (49.8%)	206 (42.9%)	0.178						
CKD 3a	104 (24.4%)	141 (21.0%)	128 (20.1%)	116 (24.2%)							
CKD 3b	86 (20.1%)	121 (18.0%)	106 (16.6%)	88 (18.3%)							
CKD 4-5	51 (11.9%)	77 (11.4%)	86 (13.5%)	70 (14.6%)							
QRS duration (ms)	162 (138 – 182)	160 (140 – 178)	156 (136 – 172)	158 (140 – 174)	0.003	0.160	***<0.001***	0.076	0.010	0.502	0.091
≤ 130 ms	76 (18.1%)	130 (18.9%)	131 (20.3%)	83 (17.5%)	0.204						
130 – 150 ms	79 (18.9%)	140 (20.4%)	154 (23.9%)	114 (24.0%)							
>150 ms	264 (63.0%)	417 (60.7%)	360 (55.8%)	278 (58.5%)							
Conduction											
Normal	28 (6.7%)	55 (8.0%)	53 (8.2%)	39 (8.2%)	<0.001	0.088	***<0.001***	***<0.001***	0.185	***<0.001***	***<0.001***
RBBB	27 (6.4%)	63 (9.2%)	71 (10.9%)	37 (7.8%)							
LBBB	285 (67.7%)	472 (68.6%)	459 (70.7%)	277 (58.2%)							
Aspecific	33 (7.8%)	47 (6.8%)	35 (5.4%)	78 (16.4%)							
Paced	48 (11.4%)	51 (7.4%)	31 (4.8%)	45 (9.5%)							
Rhythm											
Sinus	319 (75.4%)	514 (74.7%)	514 (79.7%)	333 (70.0%)	<0.001	0.068	***<0.001***	0.117	0.015	0.074	***<0.001***
AF	62 (14.7%)	127 (18.5%)	108 (16.7%)	94 (19.8%)							
paced	42 (9.9%)	47 (6.8%)	23 (3.6%)	49 (10.3%)							
ACE / ARB / ARNI	393 (91.6%)	606 (88.0%)	571 (87.0%)	375 (77.6%)	<0.001	0.054	0.019	***<0.001***	0.614	***<0.001***	***<0.001***
BB	366 (85.3%)	596 (86.5%)	549 (83.7%)	408 (84.5%)	0.523						
MRA	245 (57.1%)	434 (63.1%)	397 (60.5%)	292 (60.5%)	0.265						
Loop diuretic	328 (78.1%)	441 (64.0%)	348 (53.3%)	302 (62.7%)	<0.001	***<0.001***	***<0.001***	***<0.001***	***<0.001***	0.637	***0.002***

Amiodarone	120 (28.0%)	144 (21.0%)	142 (21.7%)	108 (22.4%)	0.041	***0.008***	0.019	0.051	0.747	0.575	0.794
Hypertension	239 (55.7%)	447 (64.7%)	522 (79.7%)	327 (67.7%)	<0.001	***0.003***	***<0.001***	***<0.001***	***<0.001***	0.284	***<0.001***
Dyslipidemia	266 (62.2%)	390 (56.7%)	467 (71.5%)	328 (68.1%)	<0.001	0.071	***0.001***	0.062	***<0.001***	***<0.001***	0.208
Stroke	49 (11.5%)	71 (10.3%)	55 (8.4%)	54 (11.4%)	0.287						
Diabetes Mellitus	112 (26.1%)	174 (25.2%)	177 (27.0%)	139 (28.7%)	0.600						

* Correction for multiple testing: p≤0.008 required for significance.

ICD–implantable cardioverter-defibrillator; ICMP–ischemic cardiomyopathy; LVEF–left ventricular ejection fraction; RBBB–right bundle branch block; LBBB–left bundle branch block

Supplementary Table S3. Final Cox proportional hazard regression model for the combined endpoint by implant period.

Variable	Hazard Ratio	95% CI	p-value
Female	0.65	0.52 – 0.81	<0.001
ICD	0.66	0.55 – 0.79	<0.001
ICMP	1.29	1.09 – 1.53	0.004
LVEF (/%)	0.98	0.97 – 0.99	0.003
NYHA			
I	reference		
II	1.80	0.86 – 3.73	0.118
III or IV	2.08	1.01 – 4.27	0.047
Renal function			
CKD 1–2	reference		
CKD 3a	1.21	0.97 – 1.51	0.099
CKD 3b	1.66	1.33 – 2.08	<0.001
CKD 4–5	2.67	2.09 – 3.41	<0.001
QRS duration			
≤ 130 ms	reference		
130 – 150 ms	0.75	0.58 – 0.98	0.032
>150 ms	0.78	0.63 – 0.97	0.026
LBBB	0.73	0.61 – 0.87	<0.001
ACE / ARB / ARNI	0.59	0.46 – 0.74	<0.001
Diabetes mellitus	1.21	1.02 – 1.44	0.032
Stroke / TIA	1.26	1.01 – 1.58	0.041
Implant period			
Period 1	reference		
Period 2	0.93	0.75 – 1.14	0.464
Period 3	0.87	0.67 – 1.13	0.288
Period 4	1.02	0.76 – 1.37	0.883

The model was stratified by implanting center and use of loop diuretics due to violation of the Schoenfeld residuals. Global Schoenfeld residuals test of the final model: p=0.382. Harrell's C-index of the final model = 0.669. ICD–implantable cardioverter-defibrillator; ICMP–ischemic cardiomyopathy; LVEF–left ventricular ejection fraction; LBBB–left bundle branch block

Supplementary Table S4. Between group comparison of demographics and clinical characteristics by rhythm and QRS morphology.

Variable	SR + LBBB	SR+ non-LBBB	AF + LBBB	AF + non-LBBB	QRS <130 ms	Overall p-value	Between group comparison*									
							SR+LBBB vs SR +non-LBBB	SR+LBBB vs AF+LBBB	SR+LBBB vs AF+non-LBBB	SR+LBBB vs QRS <130ms	SR+non-LBBB vs AF+LBBB	SR+non-LBBB vs AF+non-LBBB	SR+non-LBBB vs QRS<130ms	AF+LBBB vs AF+non-LBBB	AF+LBBB vs QRS<130ms	AF+non-LBBB vs QRS<130ms
N	986	170	149	49	310											
Age implant (y)	69.3 (60.9 – 75.8)	68.4 (59.7 – 75.3)	74.0 (67.4 – 80.2)	72.8 (66.5 – 80.0)	67.7 (59.6 – 75.0)	<0.001	0.209	***<0.001***	0.008	0.071	***<0.001***	***0.003***	0.891	0.375	***<0.001***	***0.001***
Female	334 (33.7%)	24 (14.1%)	26 (17.3%)	5 (10.2%)	65 (20.8%)	<0.001	***<0.001***	***<0.001***	***<0.001***	***<0.001***	0.429	0.476	0.069	0.232	0.376	0.080
ICD	694 (70.1%)	132 (77.7%)	77 (51.3%)	32 (65.3%)	241 (77.2%)	<0.001	0.045	***<0.001***	0.475	0.014	***<0.001***	0.079	0.919	0.088	***<0.001***	0.070
Upgrade	147 (14.9%)	58 (34.1%)	47 (31.3%)	15 (30.6%)	50 (16.0%)	<0001	***<0.001***	***<0.001***	***0.003***	0.613	0.597	0.647	***<0.001***	0.925	***<0.001***	0.013
Epicardial	47 (4.8%)	9 (5.3%)	10 (6.8%)	1 (2.0%)	12 (3.9%)	0.604										
ICMP	364 (36.8%)	99 (58.6%)	73 (48.7%)	24 (49.0%)	157 (50.7%)	<0.001	***<0.001***	***0.005***	0.086	***<0.001***	0.076	0.233	0.096	0.970	0.691	0.828
LVEF (%)	25.0 (20.0 – 30.0)	25.0 (20.0 – 30.0	25.0 (20.0 – 30.0	29.0 (25.0 – 30.0)	25.0 (20.0 – 30.0	0.309										
NYHA																
I	35 (3.6%)	9 (5.4%)	5 (3.4%)	0 (0.0%)	9 (2.9%)	0.214										
II	293 (29.8%)	52 (51.1%)	32 (21.5%)	13 (27.7%)	91 (29.6%)											
III	621 (63.1%)	98 (58.7%)	100 (67.1%)	33 (77.2%)	191 (62.0%)											
IV	36 (3.7%)	8 (4.8%)	12 (8.1%)	1 (2.1%)	17 (5.5%)											
eGFR (mL/min)	60.6 (43.0 – 77.3)	55.0 (40.6 – 70.7)	48.7 (32.9 – 61.9)	47.2 (35.8 – 60.4)	59.6 (41.6 – 74.2)	<0.001	0.036	***<0.001***	***<0.001***	0.332	***0.003***	0.041	0.259	0.863	***<0.001***	***0.004***
CKD 1–2	500 (51.3%)	72 (42.9%)	44 (29.5%)	13 (27.7%)	151 (49.4%)	<0.001	0.167	***<0.001***	0.008	***0.003***	0.047	0.101	0.151	0.267	***<0.001***	***<0.001***
CKD 3a	201 (20.6%)	43 (25.6%)	40 (26.9%)	12 (25.5%)	68 (22.2%)											

CKD 3b	176 (18.1%)	31 (18.5%)	32 (21.5%)	16 (34.0%)	37 (12.1%)											
CKD 4–5	97 (10.0%)	22 (13.1%)	33 (22.2%)	6 (12.8%)	50 (16.3%)											
ACE / ARB / ARNI	886 (89.5%)	138 (81.2%)	122 (81.3%)	43 (87.8%)	277 (88.8%)	0.003	***0.002***	***0.004***	0.699	0.722	0.971	0.284	0.021	0.300	0.029	0.833
BB	865 (87.4%)	143 (84.1%)	130 (86.7%)	40 (81.6%)	276 (88.5%)	0.521										
MRA	651 (65.8%)	105 (61.8%)	98 (65.3%)	23 (46.9%)	208 (66.7%)	0.075										
Loop diuretic	594 (60.4%)	123 (72.4%)	113 (75.3%)	35 (71.4%)	217 (70.0%)	<0.001	***0.003***	***<0.001***	0.123	***0.003***	0.545	0.899	0.553	0.587	0.215	0.814
Amiodarone	205 (20.7%)	55 (32.4%)	41 (27.3%)	11 (22.5%)	69 (22.2%)	0.011	***<0.001***	0.066	0.769	0.577	0.328	0.183	0.015	0.499	0.224	0.967
Hypertension	635 (64.2%)	118 (69.4%)	120 (80.0%)	33 (67.4%)	211 (67.6%)	0.004	0.189	***<0.001***	0.654	0.269	0.030	0.783	0.688	0.068	0.006	0.969
Dyslipidemia	613 (62.1%)	121 (71.2%)	105 (70.0%)	28 (57.1%)	198 (63.5%)	0.069										
Stroke	102 (10.4%)	14 (8.2%)	23 (15.4%)	4 (8.2%)	19 (6.1%)	0.025	0.396	0.065	0.621	0.025	0.045	0.987	0.378	0.198	***0.001***	0.585
Diabetes Mellitus	254 (25.7%)	57 (33.5%)	35 (23.3%)	15 (30.6%)	91 (29.2%)	0.155										

* Correction for multiple testing: p≤0.005 required for significance

LBBB–left bundle branch block; ICD–implantable cardioverter-defibrillator; ICMP–ischemic cardiomyopathy; LVEF–left ventricular ejection fraction

Supplementary Table S5. Detailed incidence and cumulative event rates for the combined endpoint by rhythm and QRS morphology and duration in patients with LVEF ≤ 35%.

Rhythm	QRS duration	Morphology	Total n	Events (%)	Incidence rate %/y (95% CI)	Cumulative event rate (%)		
						1 y	3 y	5y
SR	>150	LBBB	730	188 (25.8%)	6.1% (5.3 – 7.1)	4.5%	16.5%	25.2%
SR	>150	Non-LBBB	113	41 (36.3%)	12.7% (9.3 – 17.2)	16.6%	29.2%	45.8%
SR	130–150	LBBB	256	58 (22.7%)	5.8% (4.5 – 7.5)	4.0%	14.3%	27.0%
SR	130–150	Non-LBBB	57	21 (36.8%)	10.5% (6.8 – 16.0)	9.2%	18.7%	38.4%
SR	<130		233	77 (33.0%)	9.8% (7.8 – 12.3)	10.2%	21.9%	36.9%
AF	>150	LBBB	96	36 (37.5%)	11.3% (8.1 – 15.7)	16.9%	29.8%	40.5%
AF	>150	Non-LBBB	26	15 (57.7%)	21.1% (12.7 – 35.0)	20.2%	44.9%	55.9%
AF	130–150	LBBB	53	20 (37.7%)	9.8% (6.3 – 15.2)	7.5%	23.9%	40.9%
AF	130–150	Non-LBBB	23	8 (34.8%)	11.1% (5.3 – 23.4)	32.0%	37.8%	37.8%
AF	<130		77	26 (33.8%)	10.2% (6.9 – 14.9)	8.0%	19.1%	49.3%

LVEF–left ventricular ejection fraction

Supplementary Table S6. Final Cox proportional hazard regression model for the combined endpoint by rhythm and QRS morphology.

Variable	Hazard Ratio	95% CI	p-value
Female	0.60	0.47 – 0.77	<0.001
ICD	0.59	0.47 – 0.73	<0.001
ICMP	1.25	1.03 – 1.53	0.027
LVEF (/%)	0.97	0.96 – 0.99	0.001
NYHA			
I	reference		
II	2.12	0.84 – 5.30	0.110
III or IV	2.28	0.92 – 5.66	0.074
Renal function			
CKD 1–2	reference		
CKD 3a	1.21	0.94 – 1.56	0.136
CKD 3b	1.59	1.22 – 2.07	0.001
CKD 4–5	2.56	1.94 – 3.38	<0.001
ACE / ARB / ARNI	0.55	0.42 – 0.72	<0.001
Diabetes mellitus	1.28	1.05 – 1.56	0.016
Stroke / TIA	1.31	1.00 – 1.72	0.047
Indication			
SR + LBBB	reference		
SR + non-LBBB	1.51	1.12 – 2.03	0.006
AF + LBBB	1.33	0.97 – 1.80	0.072
AF + non-LBBB	2.08	1.30 – 3.33	0.002
QRS < 130 ms	1.64	1.29 – 2.09	0.002

The model was stratified by implanting center and use of loop diuretics due to violation of the Schoenfeld residuals. Global Schoenfeld residuals test of the final model: p=0.118. Harrell's C-index of the final model = 0.683. ICD–implantable cardioverter-defibrillator; ICMP–ischemic cardiomyopathy; LVEF–left ventricular ejection fraction

Supplementary Table S7. QRS duration categorized

Variable	QRS ≤ 130 ms	QRS 130 – 150 ms	QRS >150 ms	p-value
N	420 (18.9%)	487 (21.9%)	1319 (59.2%)	
Age implant	69.5 (61.0 – 75.5)	70.3 (61.4 – 76.9)	70.7 (62.5 – 77.2)	0.039
Female	97 (23.1%)	163 (33.5%)	327 (24.8%)	<0.001
ICD	277 (66.0%)	311 (63.9%)	828 (62.8%)	0.506
Upgrade	73 (17.4%)	82 (16.8%)	436 (33.1%)	<0.001
Epicardial	19 (4.6%)	26 (5.3%)	72 (5.5%)	0.747
ICMP	202 (48.4%)	201 (41.5%)	546 (41.6%)	0.037
NYHA				
I	18 (4.4%)	15 (3.1%)	52 (4.0%)	0.373
II	135 (32.7%)	165 (34.2%)	388 (29.8%)	
III	241 (58.4%)	280 (58.1%)	815 (62.6%)	
IV	19 (4.6%)	22 (4.6%)	47 (3.6%)	
LVEF	28.5 (21.0 – 35.0)	29.0 (23.0 – 35.0)	26.5 (20.0 – 33.0)	0.005
≤ 35%	323 (78.8%)	405 (84.6%)	1126 (86.6%)	<0.001
35% – 50%	64 (15.6%)	65 (13.6%)	161 (12.4%)	
>50 %	23 (5.6%)	9 (1.9%)	13 (1.0%)	
eGFR	60.2 (42.5 – 77.2)	57.7 (42.1 – 73.6)	56.9 (40.1 – 74.3)	0.134
CKD 1–2	207 (50.4%)	224 (46.4%)	598 (46.2%)	0.139
CKD 3a	90 (21.9%)	114 (23.6%)	278 (21.5%)	
CKD 3b	55 (13.4%)	89 (18.4%)	252 (19.5%)	
CKD 4–5	59 (14.4%)	56 (11.6%)	166 (12.8%)	
Center				
UZL	107 (25.5%)	167 (34.3%)	492 (37.3%)	0.001
USZ	140 (33.3%)	147 (30.2%)	370 (28.1%)	
ZOL	173 (41.2%)	173 (35.5%)	457 (34.7%)	
QRS	116 (101 – 124)	142 (138 – 146)	172 (162 – 186)	<0.001
Conduction				
Normal	174 (41.6%)	0 (0.0%)	0 (0.0%)	<0.001
RBBB	25 (6.0%)	57 (11.7%)	115 (8.7%)	
LBBB	155 (37.1%)	364 (74.7%)	972 (73.8%)	
Aspecific	53 (12.7%)	52 (10.7%)	88 (6.7%)	
Paced	11 (2.6%)	14 (2.9%)	142 (10.8%)	
Rhythm				
Sinus	296 (70.5%)	376 (77.4%)	1002 (76.1%)	<0.001
AF	112 (26.7%)	96 (19.8%)	181 (13.8%)	
paced	12 (2.9%)	14 (2.9%)	133 (10.1%)	
ACE / ARB / ARNI	360 (85.7%)	419 (86.0%)	1136 (86.2%)	0.970
BB	352 (83.8%)	413 (84.8%)	1124 (85.3%)	0.763
MRA	260 (61.9%)	311 (63.9%)	774 (58.8%)	0.117
Loop diuretic	266 (63.6%)	296 (61.0%)	836 (63.8%)	0.551
Amiodarone	85 (20.3%)	110 (22.6%)	304 (23.1%)	0.501
Hypertension	294 (70.0%)	335 (68.9%)	878 (66.7%)	0.370
Dyslipidemia	261 (62.3%)	303 (62.4%)	863 (65.8%)	0.248
Stroke	32 (7.7%)	54 (11.2%)	138 (10.5%)	0.165
Diabetes Mellitus	120 (28.6%)	147 (30.2%)	324 (24.6%)	0.033

ICD–implantable cardioverter-defibrillator; ICMP–ischemic cardiomyopathy; LVEF–left ventricular ejection fraction; RBBB–right bundle branch block; LBBB–left bundle branch block
